# Analysis of siblings’ relationship and parenting style using structure modelling approach

**DOI:** 10.1371/journal.pone.0281266

**Published:** 2023-02-02

**Authors:** Kristýna Krejčová, Hana Chýlová, Pavla Rymešová

**Affiliations:** Department of Psychology, Faculty of Economy and Management, Czech University of Life Sciences, Prague, Czech Republic; The Hong Kong Polytechnic University, HONG KONG

## Abstract

Relationships among siblings play a crucial role in early socialization and further social development. Siblings inherently belong to the system of family relationships; their observation without involving parent-child bonds, is to some extent, generalization. Therefore, our research analyzes with mutual connections between parenting styles and the characteristics of siblings’ relationships in a family using a quantitative design of data collection and processing. The outputs from the Sibling Relationship Questionnaire and Questionnaire of Parenting Styles in a Family were collected from 264 bachelor’s students. The study found the overall associations of positive emotional relationships and freedom provided by parents with generally desirable characteristics of siblings’ bonds and vice versa, and associations between negative emotional relationships and requirements with negatively perceived traits of siblings’ bonds. The unexpected outcome of our research is that it revealed more connections between paternal parenting styles and the characteristics of sibling relationships than with those of maternal parenting styles. To reflect the complexity of these relationships more precisely, complex structural equation models were created and successfully adjusted.

## Introduction

The family is considered a group for primary socialization that substantially determines the development of human personality. Many studies have examined the significance of parenting style [1]. Although siblings’ interactions enjoy much less scientific attention, the systemic approach in family studies highlights that we cannot fully understand the behavior of the family system without involving siblings [2]. Similarly, we cannot study siblings’ relationships without considering their interactions with their parents. Therefore, our research aimed to examine the mutual influences between the characteristics of siblings’ relationships and parental styles. We realized that family relationships are dynamic, and the research observes only a particular stage in their development. We intentionally dealt with these connections in early adulthood, since these stadia provide an opportunity to explore the long-term effects of social backgrounds [3].

### Quality of siblings’ relationship

Among individuals, siblings form the longest close relationship with each other. Although bond quality may greatly vary, it considerably influences personality development. Adler’s classical work [4] laid the foundation for perceiving siblings’ relationships through family constellations. Subsequent research confirmed the role of constellation variables. According to Furman and Buhrmester [5], older siblings nurture younger ones more; however, they also perceive more rivalry and dominance over the sibling, especially in the case of same-sex individuals. Young individuals admire their siblings more and perceive them as being more favored by their parents. Nevertheless, older siblings describe their relationships with siblings as less harmonious [6] and more conflictual [7].

Considering the gender specifics in the siblings’ constellation, the sisters’ relationships appear to be warmer, but also more conflictual [7, 8]. Sisters’ relationship with each other also includes the aspect of more empathic care [9]. Even though the dyad of mixed-gender siblings’ is generally less conflictual than same-sex relationships [10], Buhrmester and Furman [6] pointed out that girls rate their relationships with sisters significantly better than with brothers. For boys, same-sex sibling preferences were analogical but insignificant.

Age-spacing is also has important. Furman and Buhrmester [5] refer to more conflicts and less admiration in narrow-spaced dyads (less than four years), whereas older children report more nurturing and caretaking behavior than those in the narrow-spaced dyads. On the other hand, narrow-spaced siblings have more intimacy in their relationships [6] and spend more time together in the same developmental stadium, which makes their relationships closer [11].

The research on constellation variables is substantial; nevertheless, the complex quality of siblings’ relationships does not seem to be primarily determined by constellation influences [5, 10]. This crucial role may be played, for instance, by personal influences [9], by the time spent together [10] or by relationships with parents [12–14].

Analogous to the whole family system, siblings’ relationships change with the development of their actors. Although the amount of leisure time among siblings varies with their age distance, and other constellational influences [15], it generally decreases in adolescence and even more in early adulthood. Consequently, siblings in this developmental stage are engage less in joint activities and generally have fewer behavioral interactions; however, they have higher involvement in emotional exchanges and warmth and show less intensity of conflict and rivalry than in adolescence [13, 16]. Nevertheless, adolescence changes the shape of siblings’ relationships, decreasing asymmetry and intensity and becoming more egalitarian. This trend may precede the modification of power-distribution in the parent-child relationship [6].

The impact of these developmental tendencies on siblings’ relationships considerably depends on their mutual age spacing which determines whether they experience developmental shifts in similar periods, which enables sharing experiences and strengthens their relationships [11]. Nevertheless, with the increasing age of siblings, even the impact of relationships with parents on siblings’ bonds changes. This impact weakens during early adulthood [16]. In contrast, siblings in early adulthood appear to be sensitive to parental differential parental treatment [8]. Parental differentiation may deepen in adulthood [17]. According to the theory in evolutionary biology theory [18], siblings tend to avoid direct conflict over parental resources. The psychological correlates of this theory were shown in a study by Feinberg et al. [19] who show that siblings with different trajectories of relationships with parents experience more warmth and maintain fewer conflictual bonds (in correspondence with a lower tendency to rivalry). From the viewpoint of evolutionary biology, differences between siblings are explained as the prevention of from lineage extinction, because variability increases the general adaptability of a generation of offspring [20].

The mutual difference between siblings can also be explained from the viewpoint of social comparison [21], which is mediated to children by their parents who tend to magnify small differences they perceive between siblings perceived in their early development [22]. According to Finzi-Dottan and Cohen [8], both too high and too low a level of similarity between siblings contributes to conflict in their relationship. The authors deduced that siblings need to be different. However, extreme dissimilarities make these bonds more conflictual.

According to Furman and Buhrmester [5], similarity and parental partiality are the fundamental characteristics of siblings’ relationships. Based on a component analysis of interviews with upper elementary school children about their perception of sibling relationships, the authors defined four crucial factors: warmth, relative status, conflict and rivalry. In their subsequent analysis, only conflict and rivalry were correlated with each other. With the Siblings Relationship Questionnaire, we measured these factors through 15 scales, including (except similarity and maternal/paternal partiality) intimacy, prosocial behavior, companionship, nurturance of/by siblings, admiration of/by siblings, affection, dominance of/by siblings, quarreling, antagonism and competition. In a subsequent study dealing with siblings’ relationships in early adulthood, Stocker et al. [10] described only three factors (warmth, conflict and rivalry), which corresponds to the fact that siblings’ bonds in this developmental stage become less asymmetrical and more egalitarian [6]. This line of research expresses the tendency to shift the study on siblings’ relationships from the simplified and superficial perspective of constellation variables to a more elaborate and meaningful view that contributes to a deeper understanding of siblings’ bonds.

### Parenting styles

Sibling’ bonds do not exist in an interactional vacuum. They are mediated and modified by relationships with other members in society, particularly when they interact with their parents both indirectly (e.g., the quality of parent-child attachment) and directly (e.g., mediation of conflict between siblings by parents) [12]. As a child’s personality develops in the frame of parenting styles, parents determine the quality of siblings’ relationships.

A substantial volume of research on parental styles is covered by the metanalytical study of Kuppens et al. [1]. Based on cluster analysis, they formulated four parenting styles: authoritative, positive authoritative, authoritarian and uninvolved, considering the positive authoritative style as optimal and authoritarian as pessimal. The authors identified two crucial parenting dimensions (parental support and behavioral control) that are analogous to the conceptualization of emotional warmth and parental control used in this study [23]. These classifications correspond with the classical conception of authoritative, authoritarian, and permissive parenting styles proposed by Baumrind [24] based on the different forms of parental control: authoritarian, authoritative, and permissive control [25].

This theory inspired other classical authors, Maccoby and Martin [26], who determined two fundamental dimensions of parenting styles: warmth and strictness. Warmth refers to the expression of positive feelings towards the child, supportive communication, acceptance, and spending time together; whereas strictness is understood as a level of control, supervision and strictness in setting norms and rules for a child [23, 26–29], which increases the overall generalizability of Baumrind’s theory [27]. A measure of warmth and strictness and their mutual combinations define the raising style of parents labelled as authoritarian (low warmth and high strictness), authoritative (high strictness and warmth), indulgent (high warmth and low strictness) and neglectful (low strictness and warmth) [26–27, 30–33].

A conceptual framework for this paper is based on the dimensions of emotional relationships and parental behavioral control, which are analogous to warmth and strictness [23]. To the previously described classical four parenting styles, Čáp [23] added specifications of inconsistent behavioral control (typical for very unstable parents) and extremely positive emotional relationships (strong identification of a child with a parent) and created a model of nine parental styles: authoritarian (negative relationship, strong or medium control), neglectful (negative relationship, weak control), pessimal (negative relationship, inconsistent control), emotionally inconsistent (one parent neglecting, second extremely caring and positive), authoritative (positive or extremely positive relationship, strong control), optimal (positive or extremely positive relationship, medium control), indulgent (positive relationship, weak control), friendly (extremely positive relationship, weak control), and inconsistent control relatively balanced by positive emotional relationship [34].

Using conceptualizations described in this research, we need to consider certain simplifications of all the classificational theories of parenting styles. General parenting styles influence parent-child interaction in the concrete form of parenting practices. The relationship between parenting styles and practices is not straightforward, and its specifications should be studied and reflected [27].

Gender specifics of parenting styles have been the subjects of many studies. De Bel, Kalmijn and van Duijn [14] revealed that regarding support exchange (mutual exchange of help in household tasks and odd jobs, advice, showing and receiving interest from each other) and the amount of contact (face-to-face, phone, email etc.) in the family, the mother-child relationship has a bigger impact on siblings than the father-child relationship. De Graaf, Hoogenboom, De Roos and Bucx [35] expected that fathers’ parenting behavior would be more contextual (determined by parents’ and children’s characteristics and the level of social support from parents, other family members, friends, neighbors and childcare/school). However, this hypothesis was only partially confirmed. Parents reported similar levels of reward, punishment and autonomy support; mothers scored higher only on affection, responsivity and explanation. According to a study of Pinquart [36], girls’ academic achievement was stronger with maternal warmth than with paternal warmth. However, similar analogical relationships were not found in boys. Generally, parents tend to spend more time with siblings of the same gender (mothers with daughters, fathers with sons), and share more activities with them. Although these findings are fruitful, the author studies maternal and paternal influences on children’s achievement separately, neglecting their interconnectedness. To deepen our understanding of family dynamics, a complex study of mutual relations and their connections is necessary. Despite the general scientific agreement about the systemic nature of family relationships, the specific processes interrelating different relationships have not received sufficient research attention [5].

### The complexity of family relations

Although siblings’ relationships in early adulthood depend less on interactions with parents, we can track the influence of a positive family atmosphere, which leads to warm relationships between siblings. Distress in the family has a destructive impact on siblings’ bonds. According to Scharf et al. [16], this effect is more complex and substantially depends on the sibling’s age. In emerging adulthood, functional (positively perceived) dependence on parents is related to more warmth and less rivalry in siblings’ relationships, whereas conflictual dependence leads to higher levels of conflict and rivalry.

Studying the subsystem of the sibling-parent-sibling triad, de Bel, Kalmijn, and van Dujin [14] revealed that even in adulthood, the conflict with either parent leads to more conflictual relationships between siblings. This study assessed the plausibility of the theoretical constructs of enhancement (positive impact of relationships with parents on siblings’ bond), compensation (of negative relationships with parents by positive relationships between siblings) and loyalty conflict (if one sibling has a positive relationship with the parent while the other has a negative relationship, it may lead to negative relationships between siblings). The findings support the enhancement theory, concluding that positive relationships with parents (i.e., with mothers) predict positive support exchanges and contact with adult siblings. Similarly, the congruence between positive relationships between a parent and a child and between siblings was confirmed by Portner and Riggs [13] who analyzed two competitive presumptions of congruence (positive impact of good relationships with parents on sibling relationships through social learning processes) and compensation (of weak relationships with parents through stronger siblings’ bonds).

Clearly, parental styles and attitudes influence the quality of sibling relationships. According to Milevsky et al. [12], authoritative and permissive parenting is associated with greater mutual support and closeness between siblings compared with authoritarian and neglectful parenting styles. However, there is a bidirectional relationship, because the parent-child interaction may also be influenced by the sibling’s context. Parental styles and practices largely depend on parents’ feelings of competence in parenthood. The psychology of family relations defines this type of self-concept as parental self-efficacy (PSE) [37]. The concrete form of parental self-efficacy has a siblings-related framework, especially for the parents of younger siblings, because it is largely influenced by their experiences with older children. In the case of adolescents, a higher level of PSE predicts promotive parenting for younger children by parents with “good” experiences with older children, meaning that they did not report behavioral difficulties with these children in adolescence. In the case of difficulties with older children, PSE does not serve as a predictor of promotive parenting in younger adolescents [38].

In our study, we did not observe the influence of the constellation variables. According to Feinberg, McHale, Crouter and Cumsille [19], structural variables such as age-spacing or gender have no impact on the interplay between parent-child relationships and siblings’ mutual relationships. Although this finding deserves further empirical validation, we concentrate on the pure influence of parental styles on siblings’ relationships.

The major goal of our current study is to extend the modest volume of research that does not observe family relations separately but analyze their interplay and mutual influences. We used the Questionnaire of Parenting Styles in a Family [39] and the Sibling Relationship Questionnaire [5]. Our intention is to follow the research on gender-specifics of parenting styles [14, 24, 36] and connect this issue with the quality of siblings’ relationships. Therefore, we formulated the following hypotheses:

H1: The emotional warmth of a mother does not influence the quality of siblings’ relationships.

H2: Mothers’ behavioral control does not influence the quality of siblings’ relationships.

H3: The emotional warmth of a father does not influence the quality of siblings’ relationships.

H4: The behavioral control of a father does not influence the quality of siblings’ relationships.

## Materials and methods

### Procedure

The present study was approved by the Ethics Committee of the Czech University of Life Sciences in Prague and conducted in accordance with the ethical principles stated in the Declaration of Helsinki (2013). All respondents were instructed and provided written consent to participate. Data were analyzed anonymously in accordance with the ethical standards of the American Psychological Association.

Our research sample was constructed using a convenience choice method and involved 264 respondents (full-time bachelor’s students of the Czech University of Life Sciences Prague). The scope of the original sample was reduced because 32 students were only children and could not complete the Sibling Relationship Questionnaire. The remaining 232 respondents in the reduced research sample comprised 134 females and 96 males. Two respondents did not specify their sex. The mean age was 20.2 years with a maximum of 29 years and a minimum of 18 years.

Considering the constellation variables, we had 155 respondents with one sibling, 51 with two siblings, 17 with three siblings, and 6 with four or more siblings (three respondents did not specify the number of siblings). The respondents received an instruction that in case of more siblings, they should fill in the Sibling Relationship Questionnaire considering their “nearest” siblings in terms of age spacing and emotional bond. A total of 101 respondents had a female sibling, and 124 had a male sibling. Eighty-two respondents had siblings of age distance up to 3 years, and as many respondents had siblings between 4 and 6 years of age distance. The remaining 68 respondents were 7 and more years far from their closest sibling.

### Measures

#### Sibling relationship questionnaire

Considering the quantitative design of our research project, we used a battery of two standardized questionnaires. The Sibling Relationship questionnaire comprises 16 scales of fundamental characteristics of siblings’ interactions assessed in 48 items (39 in the shortened version). The composition of the scales was determined according to crucial categories stemming from interviews with respondents about the perceived qualities of their siblings’ relationships. After the initial coding process (three research assistants sorted the elements of the interview into separate categories), the authors created a coding manual that was used for the validation of the scale—all the interviews were processed by two naive assistants with the interrater agreement expressed by Cohen kappa coefficient between 0.66 and 1.00 by all scales. Furthermore, a questionnaire containing three Likert-type items on each scale was created. In the psychometric analysis of the subsequent validation study, the internal consistency coefficient (Cronbach’s alpha) exceeded .70 for almost all scales (with the exception of competition with Cronbach’s alpha .63). The mean test-retest reliability for all scales was r = .71 [5].

The scale Prosocial behavior covers elements of siblings’ interactions such as sharing and doing nice things for each other. Affection refers to mutual caring and love between siblings; Companionship is related to cooperation and having fun together; Similarity covers a closeness of interests and preferences; Intimacy is related to sharing of secrets and deepest feelings. Admiration of/by sibling expresses mutual respect and pride on each other. Aspects of siblings’ mutual help and teaching new things are covered by Nurturance of/by sibling. The Dominance of/by sibling is understood as commanding or even forcing to do things. Paternal/maternal partiality is related to the perception of parental preference among siblings. Competition is understood as the tendency to win over other siblings. Antagonism refers to mutual offenses and means to each other. Quarreling is related to frequent conflicts and lack of mutual agreement [5, 6].

In the subsequent principal component analysis, four factors were identified: Warmth/Closeness, Relative Status/Power, Conflict and Rivalry The closest factors were Conflict and Rivalry, the mutual correlations of the other factors were minimal. Regarding the constellation effect, Warmth/Closeness was identified as more intensive by narrow-spaced, same-sex siblings. Relative Status/Power showed a greater influence over younger siblings (especially by the wide-spaced sibs). Conflict is greater by wide-spaced siblings, whereas Rivalry is more intensive by younger siblings [5].

In our research, we used the translated version of the shortened questionnaire with an age-related adaptation and a slight modification by Parental partiality, assessed only in the terms of better treatment of the respondent in comparison to their siblings. This methodological change was due to the unification of the scoring system, which helped to minimize mistakes in response processes and gain more reliable findings. Therefore, both scales of Parental partiality refer only to the parental preference of respondents and not to the possible preference of their siblings.

#### Questionnaire of parenting styles in a family

The standardized questionnaire for the identification of parenting styles in a family was designed for children and young adults based on the two-dimensional model of nine parenting styles [23]. The first dimension was represented by the emotional relationship measured on two scales: the level of positive and negative emotional relationships. The second dimension consists of parental control, represented by the scales of requirements (high control, strictness, and supervision) and freedom (low control, more space for children’s choice of activities, and so on). On this basis, a model of nine parenting styles was created—possible combinations of positive, neutral, and negative relationship with strong, medium, weak or inconsistent parental control. The questionnaire comprised 40 items assessed on a 3-grade Likert-type scale (“yes-partially-no”). Each of the four measures of parenting style (positive emotional relationship, negative emotional relationship, requirements, freedom) was covered by 10 items. Respondents were asked to assess the raising style of each parent separately.

### Analytic plan

The first step in the data processing was descriptive analysis. Further, we assessed the normality of our data using the Shapiro-Wilk test of normality, which is widely recommended for its statistical power [40]. To test the hypotheses about the Sibling relationship and Parenting styles, we used a measure of linear relationships (Pearson’s correlation or Spearman’s rank correlation for its robustness [41], depending on the tested normality of our data). The data were processed with the statistical software IBM SPSS Statistics, version 27, and all the results are in the respective section.

Apart from standard multidimensional statistical methods, we used the structural equation modelling methodology provided by IBM SPSS Amos v5 software to more precisely reflect the complexity of relationships between parenting styles and the quality of siblings’ relationships. The model fit will be evaluated on the basis of chi-square goodness-of fit statistics, comparative fit index (CFI), Tucker-Lewis index (TLI) and root mean square error of approximation (RMSEA). Moreover, two comparative fit indices for the tested and saturated model were considered: Akaike information criterion (AIC) and Bayesian information criterion (BIC).

## Results

### Descriptive statistics

Our descriptive analysis covered the measures of central tendency, variability, and dispersion, including a confidence interval for the mean (see Tables 1 and 2). The descriptive statistics already yielded some interesting findings for scales. The Sibling relation quality scales (Table 1) displayed the highest average levels of all the scales forthe Prosocial behavior (9.62) as well as for the Nurturance of sibling (9.02), with the lowest levels of the Antagonism (4.57) and Competition (4.73) between siblings, which could be seen as an indicator of frequent harmonic relationships.

**Table 1:** Descriptive statistics—Sibling relationship quality scales.

	N	Min.	Max.	Mean	Std. Error	95% Confidence Interval for Mean		Std. Deviation	Variance
						Lower Bound	Upper Bound		
**Prosocial**	224	3	15	9.62	.190	9.26	9.99	2.837	8.048
**Maternal Part**	224	0	15	6.68	.200	6.22	7.02	2.997	8.981
**Nurturance of s**	224	3	15	9.02	.214	8.58	9.42	3.197	10.219
**Nurturance by s**	224	2	15	7.94	.208	7.59	8.43	3.120	9.732
**Dominance of s**	224	2	15	6.57	.206	6.13	6.93	3.087	9.529
**Dominance by s**	224	2	14	6.31	.175	6.00	6.71	2.612	6.824
**Paternal Part**	224	0	15	6.14	.219	5.82	6.69	3.274	10.721
**Affection**	224	2	10	7.50	.124	7.26	7.76	1.861	3.462
**Companionship**	224	2	10	6.10	.139	5.84	6.40	2.084	4.344
**Antagonism**	224	1	10	4.57	.149	4.29	4.88	2.224	4.946
**Similarity**	224	1	10	6.10	.146	5.84	6.42	2.180	4.753
**Intimacy**	224	1	10	5.38	.167	5.07	5.74	2.504	6.271
**Competition**	224	1	10	4.73	.158	4.38	4.99	2.360	5.571
**Admiration of s**	224	2	10	6.91	.142	6.62	7.19	2.131	4.539
**Admiration by s**	224	2	10	6.54	.143	6.25	6.82	2.136	4.563
**Quarreling**	224	1	10	5.54	.153	5.25	5.86	2.288	5.236

(Own calculation, 2021)

**Table 2:** Descriptive statistics—Parenting styles.

	N	Min.	Max.	Mean	Std. Error	95% Confidence Interval for Mean		Std. Deviation	Variance
						Lower Bound	Upper Bound		
**Mother Emotional +**	224	14	30	26.79	.232	26.32	27.25	3.472	12.053
**Mother Emotional -**	224	7	28	14.32	.281	13.74	14.84	4.203	17.661
**Mother’s Requirements**	224	7	29	17.75	.301	17.21	18.40	4.510	20.343
**Freedom/ Mother**	224	12	28	20.45	.233	19.94	20.87	3.484	12.141
**Father Emotional +**	216	0	30	24.47	.373	23.74	25.21	5.487	30.111
**Father Emotional -**	224	0	30	13.86	.357	13.74	15.00	5.339	28.500
**Father’s Requirements**	224	0	29	16.33	.393	16.25	17.61	5.876	34.526
**Freedom/Father**	224	0	28	19.27	.386	19.38	20.59	5.772	33.320

(Own calculation, 2021)

From the mean of the reported parenting styles, it can be seen (Table 2) that the highest levels are the mother’s emotional positivity (26.79) followed closely by the father’s emotional positivity (24.47) accompanied by the freedom from the mother (20.45) and father (19.27), which could also be conceded as a sign of a positive relationship with the parents (see Table 2).

Furthermore, higher average levels of all pursued characteristics of the parenting style of mothers can be detected than those of the fathers, which seems to prove higher level of involvement as well as control in the upbringing of the children.

To explore the nature of the data, specifically, to examine the normality of the distribution of the variables, the Shapiro-Wilk test of normality was computed. In all cases, the variables were not found to be normally distributed (Table 3). Therefore, we reject the hypothesis of a normal distribution of the variable in all cases (p<0.01).

**Table 3:** Test of normality.

	Statistic	Shapiro-Wilk d*f*	Sig.
**Prosocial**	.977	216	.001
**Maternal partiality**	.954	216	<0.001
**Nurturance of sibling**	.968	216	<0.001
**Nurturance by sibling**	.971	216	<0.001
**Dominance of sibling**	.922	216	<0.001
**Dominance by sibling**	.933	216	<0.001
**Paternal Partiality**	.908	216	<0.001
**Affection**	.929	216	<0.001
**Companionship**	.964	216	<0.001
**Antagonism**	.918	216	<0.001
**Similarity**	.961	216	<0.001
**Intimacy**	.934	216	<0.001
**Competition**	.925	216	<0.001
**Admiration of sibling**	.943	216	<0.001
**Admiration by sibling**	.955	216	<0.001
**Quarreling**	.948	216	<0.001
**Mother Emotional +**	.855	216	<0.001
**Mother Emotional -**	.903	216	<0.001
**Mother’s Requirements**	.977	216	.001
**Freedom/ Mother**	.983	216	.009
**Father Emotional +**	.865	216	<0.001
**Father Emotional -**	.916	216	<0.001
**Father’s Requirements**	.963	216	<0.001
**Freedom/ Father**	.933	216	<0.001

(Own calculation, 2021)

### Testing of the hypotheses

H1: Emotional relationships with mothers do not influence the quality of siblings’ relationships.

The data analysis determined four scales that were positively correlated in terms of statistical significance with positive emotional relationships with the mothers. A stronger effect was found in the Admiration by sibling, Affection, Companionship and Prosocial behavior, and a significance at the 0.05 level was found in the Intimacy, Dominance, and Admiration of sibling.

Conversely, negative emotional relationships with mothers were positively associated with Competition and Maternal Partiality in terms of a weak positive correlation, and Admiration by sibling and Affection in terms of a weak negative correlation. A slightly stronger effect was observed only for Paternal partiality (see Table 4).

**Table 4:** Correlations of mother’s emotional relationship with siblings’ characteristics.

Spearman’s rho		Prosoc	Maternal Part.	Nurt. of S.	Nurt. by S.	Dom of S.	Dom by S.	Paternal Part	Affection	Companion.	Antagonism	Similarity	Intimacy	Competition	Admir. of S	Admir of by S	Quarreling
Mother Emotional +	CC	.176**	-.009	.105	.073	.129*	.040	-.062	.180**	.172**	.035	.052	.140*	.023	.116*	.233**	.042
	Sig	.004	.444	.059	.138	.027	.274	.179	.003	.005	.303	.218	.018	.369	.041	.000	.265
	N	224	224	224	224	224	224	224	224	224	224	224	224	224	224	224	224
Mother Emotional -	CC	-.049	.151*	.019	.028	.012	.057	.170**	-.144*	-.081	.090	-.025	-.102	.114*	-.068	-.139*	.061
	Sig	.231	.012	.389	.341	.429	.196	.005	.016	.113	.090	.356	.064	.045	.156	.019	.183
	N	224	224	224	224	224	224	224	224	224	224	224	224	224	224	224	224

** Correlation is significant at the 0.01 level.

* Correlation is significant at the 0.05 level (2-tailed).

(Own calculation, 2021)

H2: Mother’s behavioral control does not influence the quality of siblings’ relationships.

In contrast to the negative emotional relationship with the mother, we found more connections of negatively perceived characteristics of siblings’ relationships with the requirements of the mother, namely the positive correlation with the scales of Competition and Quarreling. Further, the data analysis showed a positive correlation at 0.05 level with Antagonism, Domination by Sibling and Paternal partiality. A negative correlation was found for Affection.

The freedom provided by a mother was weakly positively associated with the Antagonism, but also with the Prosocial behavior and Affection. In terms of significance at the 0.01 level, we detected a positive influence on Admiration and Nurturance by sibling (see Table 5).

**Table 5:** Correlations of mother’s behavioral control with siblings’ characteristics.

Spearman’s rho		Prosoc.	Maternal Part.	Nurt. of S.	Nurt. by S.	Dom. of S.	Dom by S.	PatenalPart.	Affection	Companion.	Antagonism	Similarity	Intimacy	Competition	Admir of S	Admir by S	Quarreling
Mother Requirements	CC	-.062	.078	.036	-.023	.068	.151*	.129*	-.115*	.009	.131*	.002	-.009	.189**	-.098	-.107	.182**
	Sig	.179	.123	.295	.364	.156	.012	.027	.043	.447	.025	.489	.446	.002	.071	.055	.003
	N	224	224	224	224	224	224	224	224	224	224	224	224	224	224	224	224
Mother Freedom	CC	.137*	.088	.107	.212**	.039	-.005	.031	.122*	.096	.140*	.030	.062	.038	.080	.175**	.014
	Sig	.020	.094	.055	.001	.279	.470	.320	.034	.076	.018	.326	.178	.285	.116	.004	.418
	N	224	224	224	224	224	224	224	224	224	224	224	224	224	224	224	224

** Correlation is significant at the 0.01 level.

* Correlation is significant at the 0.05 level (2-tailed).

(Own calculation, 2021)

H3: The emotional relationship of a father does not influence the quality of siblings’ relationships.

Regarding the positive relationship with a father, our data analysis detected significant positive connections with the Similarity, Intimacy and Nurturance of the sibling. The more substantial influence was determined by the Prosocial behavior, Affection, Companionship, Nurturance by sibling and Admiration “of” as well as “by” sibling.

Contrastingly, the negative emotional relationships were positively associated with Paternal partiality, Quarreling, and Antagonism. The correlation with Admiration by sibling was substantial but negative. We found a positive correlation with significance at the 0.05 level also with Competition, Dominance by sibling and Admiration of sibling. In addition, there was a negative correlation with the Affection Scale (see Table 6).

**Table 6:** Correlations of father’s emotional relationship with siblings’ characteristics.

Spearman’s rho		Prosoc	Maternal Part.	Nurt. of S.	Nurt. by S.	Dom. of S.	Dom by S.	Paternal Part	Affection	Companion.	Antagonism	Similarity	Intimacy	Competition	Adm. of S	Adm. by S	Quarreling
Father Emotional +	CC	.190**	.026	.158*	.177**	.060	-.014	.034	.175**	.219**	-.072	.129*	.131*	.003	.225**	.280**	-.080
	Sig	.003	.351	.010	.005	.190	.420	.308	.005	.001	.147	.030	.027	.482	.000	.000	.122
	N	224	224	224	224	224	224	224	224	224	224	224	224	224	224	224	224
Father Emotional -	CC	-.063	.068	-.025	-.041	.088	.112*	.231**	-.149*	-.094	.217**	-.062	.007	.143*	-.131*	-.172**	.169**
	Sig	.174	.156	.358	.270	.096	.047	.000	.013	.080	.001	.178	.456	.016	.025	.005	.006
	N	224	224	224	224	224	224	224	224	224	224	224	224	224	224	224	224

** Correlation is significant at the 0.01 level.

* Correlation is significant at the 0.05 level (2-tailed).

(Own calculation, 2021)

H4: The behavioral control of a father does not influence the quality of siblings’ relationship.

Similar to those of mothers, fathers’ requirements were generally positively connected with the negatively perceived characteristics of siblings’ relationships. Our data analysis detected significant differences in the Paternal partiality, Antagonism, Competition, Domination by sibling and Quarreling. A weaker influence was found in the Domination of sibling; moreover, we detected a weak negative correlation in Admiration “of” and “by” sibling and Affection.

The level of freedom provided by a father was positively associated with the Nurturance by sibling, Affection and Admiration “of” as well as “by” sibling. There is also a significance at the 0.05 level in Intimacy (see Table 7).

**Table 7:** Correlations of father’s behavioral control with siblings’ characteristics.

Spearman‘s rho		Prosoc	Maternal Part.	Nurt. Of S.	Nurt. By S.	Dom. of S.	Dom by S.	Paternal Part	Affection	Companion.	Antagonism	Similarity	Intimacy	Competition	Adm. of S	Adm. by S	Quarreling
Father Requirements	CC	-.092	.065	-.005	-.009	.111*	.177**	.246**	-.131*	-.085	.209**	-.079	-.010	.187**	-.113*	-.136*	.205**
	Sig	.084	.168	.471	.445	.049	.004	.000	.025	.103	.001	.120	.439	.003	.046	.021	.001
	N	224	224	224	224	224	224	224	224	224	224	224	224	224	224	224	224
Father Freedom	CC	.096	.010	.053	.189**	-.082	.037	.021	.167**	.108	.011	.071	.111*	-.044	.170**	.198**	-.015
	Sig	.076	.439	.213	.002	.110	.289	.375	.006	.054	.435	.145	.048	.257	.005	.001	.411
	N	224	224	224	224	224	224	224	224	224	224	224	224	224	224	224	224

** Correlation is significant at the 0.01 level.

* Correlation is significant at the 0.05 level (2-tailed).

(Own calculation, 2021)

Standard correlation coefficients between .10 and .29 represent a small association [42]; therefore, the effect size of our isolated findings is small. One cannot expect very strong correlations between the two variables in such a complex dynamics system as the family. Nevertheless, the importance of our findings lies in the overall positivity of siblings’ characteristics associated with positive features of parent-child relationships and in differences between mothers’ and fathers’ influences.

### Structural equation model

**Fig 1:**
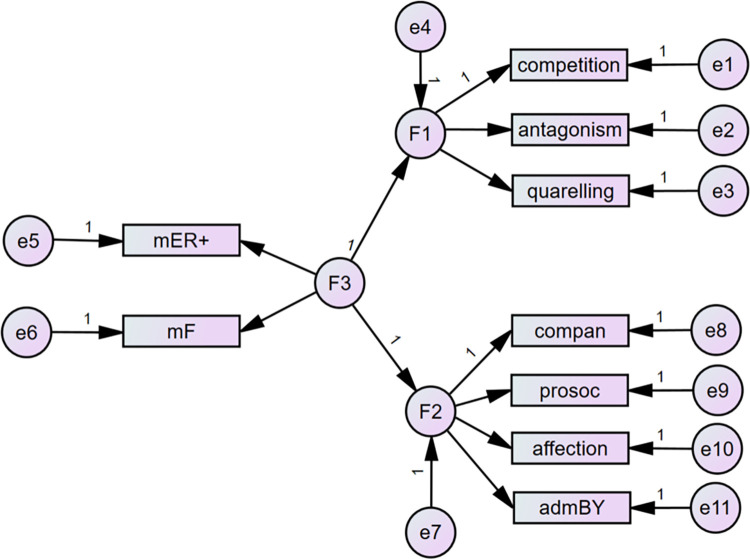
Results for theoretical model I relating maternal Warmth/freedom (F3) to siblings’ Warmth/Closeness (F2) and siblings’ Conflict (F1) with standardized coefficients. ER = emotional relationship; F = freedom; compan = companionship; prosoc = prosociality; admBY = admiration by sibling. Source: IBM SPSS Amos.

**Fig 2:**
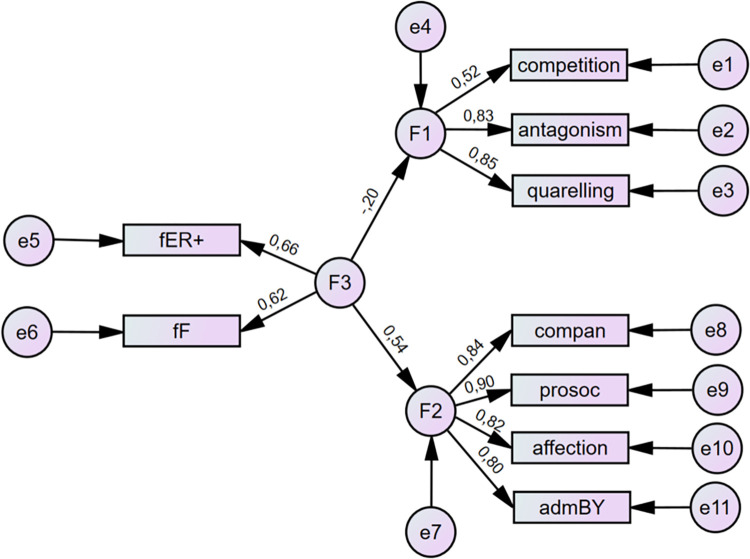
Results for theoretical model II relating paternal Warmth/freedom (F3) to siblings’ Warmth/Closeness (F2) and siblings’ Conflict (F1) with standardized coefficients. ER = emotional relationship; F = freedom; compan = companionship; prosoc = prosociality; admBY = admiration by sibling. Source: IBM SPSS Amos.

As standard multidimensional statistical methods do not mirror the complexity of observed variables and their relationships, structural equation modeling was applied. In the two-step process, we used confirmatory factor analysis (CFA) of the sibling’s relationships characteristics that were proved as significant in the previous analysis of correlations with the dimensions of parental styles. Further, the direct influence of parental style was tested. Based on these analyses, the complex structural model was developed in two versions for mothers and fathers (see Figs 1 and 2). The need for the separation of both parents came from the analysis of model fit indices and is understandable given the small or modest values of correlation between mothers and fathers’ parental styles.

**Fig 3:**
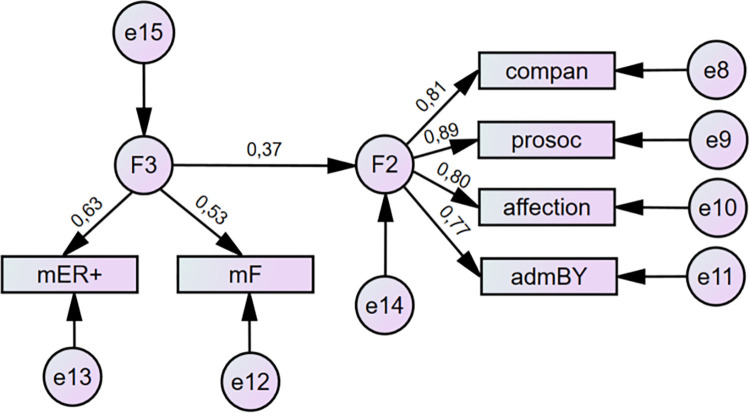
Results for theoretical model III relating maternal Warmth/freedom (F3) to siblings’ Warmth/Closeness (F1) with standardized coefficients. ER = emotional relationship; F = freedom; compan = companionship; prosoc = prosociality; admBY = admiration by sibling. Source: IBM SPSS Amos.

**Fig 4:**
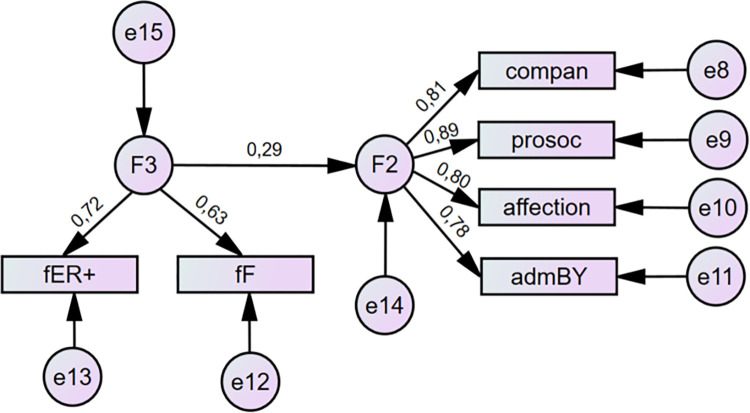
Results for theoretical model IV relating paternal Warmth/freedom (F3) to siblings’ Warmth/Closeness (F2) with standardized coefficients. ER = emotional relationship; F = freedom; compan = companionship; prosoc = prosociality; admBY = admiration by sibling. Source: IBM SPSS Amos.

According to all conventionally used SEM criteria, except RMSEA, both versions of the model set the data well. From the viewpoint of the standard statistical method, the chi-square tests of both versions proved that rejection based on the data was not possible. In both cases, the fit indices reflected a well-fit model with CFI and TLI above .95. The AIC and BIC indices proved the good comparability of the tested models with the saturated models (see Table 8). The only unsatisfactory indicator was RMSEA in the range of 0.08–0.1 which is assessed as mediocre [43]. Therefore, we continued with the analysis of the model, excluding the negative components of siblings’ relationships because of the low regression coefficient (see Figs 3 and 4). RMSEA and other indicators show that this correction was successful (see Table 8). Apparently, the same correction was not relevant in the negative dimension (relationship between parental styles and negative characteristics of siblings’ relationships) as the regression coefficient remained low.

**Table 8:** Fit indices of models.

							Our model		Saturated model	
	χ2	*df*	*p*	CFI	TLI	RMSEA	AIC	BIC	AIC	BIC
Model I	75.56	26	<0.001	0.935	0.909	0.095	113.562	177.426	90	241.258
Model II	75.116	26	<0.001	0.936	0.912	0.094	113.116	176.981	90	241.258
Model III	7.988	8	0.435	1	1	0	33.988	77.685	42	112.587
Model IV	15.14	8	0.056	0.987	0.975	0.065	41.14	84.836	42	112.587

Notes: CFI = comparative fit index; TLI = Tucker-Lewis index; RMSEA = root mean square error of approximation; AIC = Akaike’s information criteria; BIC = Bayes information criteria.

(Own calculation, 2021)

## Discussion and conclusions

Using the data from Sibling Relationship Questionnaire [5] and Questionnaire of Parenting Styles [23], the results are based on responses from ‘children as’ part of the family system. In a study by Howea, Karosa, and Aquan-Asseeb [44], children and mothers agreed on the perception of warmth in siblings’ relationships; however, the authors revealed different interpretations of aversive interaction. Mothers interpreted as antagonism what children assigned as rivalry and competition. Considering the one-sidedness of our research subject, we aimed to target the observation of young adults’ point of view on family relationships, despite its subjectivity.

Family relationships have their dynamics. From this perspective, our research observes a certain stage of their development when a large number of our respondents are separated from their families. We intentionally chose this stage because it enabled us to observe the long-term effects of social backgrounds [3]. For example, an extremely positive emotional relationship and moderate -to -weak parental control predict higher self–esteem in teenagers [45]. Similarly, perceived parenting is significantly correlated with pubescent self-concept [46]. Gender differences in perceived parenting appeared only in mothers, in the negative emotional component and in the component of demands, with significantly higher scores for girls. A gender difference in favor of the concept of boys can be seen in the overall pubescent self-concept.

Our data analysis indicates various influences of parenting styles on the quality of sibling relationships. Based on the two-dimensional model [23], we may assume that the positive emotional relationship positively correlates with positively perceived characteristics of siblings’ bond, meaning Admiration by sibling, Affection, Companionship and Prosocial behavior in mothers and the Prosocial behavior, Affection, Companionship, Nurturance by sibling and Admiration “of” as well as “by” sibling in fathers (considering only a stronger level of correlation). This finding justifies the role of a positive bond with parents in creating desirable sibling relationships and vice versa [5, 12–14].

Similar to the positive emotional relationship, we identified a connection between the desirable characteristics of siblings’ bonds and the freedom component of behavioral control. For mothers, freedom positively correlates with Admiration and Nurturance by sibling. The freedom provided by fathers is associated with the Nurturance by sibling, Affection and Admiration ‘of’ as well as ‘by’ sibling. Apparently, the freedom component correlates with Admiration and Nurturance by sibling in case of both parents. This finding leads to the constellation’s specifics in siblings’ relationship because perceived nurturance by sibling is more typical for younger siblings [5], as is the provision of freedom by parents. Participants in the study of Gozu and Newman [47] evaluated parental treatment as less fair if they received slightly more control than their siblings did.

Analogically, the negative emotional relationship with parents in our research sample is connected with negatively perceived characteristics of siblings’ bonds, namely with Paternal partiality by both parents and with Quarreling and Antagonism by fathers. Moreover, there was a significant negative correlation between Admiration by sibling. Again, there may be a self-reinforcing effect of perceived problems in siblings’ bonds that may elicit negativity in the relationship between parents and their children. These processes generally contribute to a negative atmosphere within a family.

The associations between the positive emotional relationship and freedom provided by both parents and sibling characteristics are also evident in the structural model of our results, considering only the more statistically relevant relationships (in terms of correlations with the dimensions of parental styles). We also tested the involvement of negatively perceived parental characteristics (negative emotional relationships and requirements), but the resulting model was too robust and lacked the required levels of the indices. In addition, this step was relatively redundant because the actual model fits the data better and involved the influence of parental styles on all relevant characteristics of siblings’ relationships. Moreover, further analysis of the model proved the best values of the indicators based on the positive characteristics of sibling relationships. Apparently, their bonds to parental styles were stronger and clearer.

Although Furman’s questionnaire refers to a four-factor model of siblings’ relationships involving Warmth/Closeness, Relative Status/Power, Conflict scores and Rivalry score [6], our complex model of relationships covers two factors of siblings’ relationships (Warmth and Conflict) corresponding to two factors of parenting styles (Kindness and Harshness). The reduction of Buhrman’s factor appeared, for example, in the study of Stocker, Lanthier and Furman [10] describing only 3 factors (Warmth, Conflict, and Rivalry).

Our factor of Conflict comprises the same scales as in Buhrmester’s research, unlike the factor of Warmth. In comparison with Buhrmester’s Warmth factor (composed of the scales of Intimacy, Prosocial Behavior, Companionship, Similarity, Admiration by Sibling, Admiration of Sibling, and Affection), our correlation analysis simplified this construct to include only Companionship, Prosocial Behavior, Affection and Admiration by Sibling. This adjustment is understandable considering the developmental stage of our respondents. As siblings grow older, characteristics such as intimacy or similarity may lose their significance with a general decrease in the intensity of the siblings’ relationship [6].

Our general observations are in agreement with the study of Scharf, Shulman and Avigad-Spitz [16], who worked with the same developmental stage of respondents as our research. The authors state that in emerging adulthood, functional (positively perceived) dependence on parents is related to more warmth and less rivalry in siblings’ relationships, whereas conflictual dependence leads to higher levels of conflict and rivalry. Similarly, Milevsky et al. [12] state that authoritative and permissive parenting is associated with greater mutual support (and greater closeness in case the of the authoritative style) between siblings compared to authoritarian and neglectful parenting styles.

The negative traits of sibling’ bonds are expected to correlate with the requirement dimension of parental control. Our data analysis determined the relationship between Competition and Quarreling in both parents. Moreover, there was a correlation between the Paternal partiality, Antagonism, and Domination by sibling in fathers. This observation is remarkable because parental control was not originally perceived as undesirable. Optimal parenting styles involve positive emotional relationships and a medium level of behavioral control [13]. Therefore, we did not expect a positive correlation of requirements with conclusively negatively perceived characteristics of siblings’ relationships or a positive connection of positive characteristics with the freedom dimension of behavioral control. Nevertheless, these results indicate a shift in parental style in the Czech Republic at the end of the 20^th^ century when the majority of our respondents were born. In the analysis of changes in parenting strategies in the Czech Republic between 1991 and 2002 [39], the level of emotional relationships remained stable, whereas parental control went through significant changes. In connection with the growing democratization of education styles in the Czech Republic, the research proved a significant decline in parental control. This change was evident in both mothers and fathers; however, it occurred mainly in the freedom component with no significant changes in the requirements.

The described transformation was also observed in other countries [30] and should also be considered from the viewpoint of cultural relevance [48]. Moreover, newer studies perceive parental control as a negative factor that leads to the manipulation of children’s thoughts and emotions to reach parents’ goals [1]. Martínez et al. [33] revealed the positive impact of parental practices based on acceptance and involvement on adolescent adjustment, and negative effect of strictness/imposition practices, which questioned the necessity of strictness in parental practices, similar to Garcia et al. [30]. Obviously, these effects are stable across a lifetime, as in the study of the relationship between parenting styles and social values [49] or other dimensions of psychosocial development [32]. According to Gimenez-Serrano et al. [31], greater parental warmth but not greater strictness of parents was beneficial for respondents in grandparents’ age in terms of well-being and good relationships with their descendants. A study by Perez-Gramaje et al. [50] revealed better indicators of adjustment in non-aggressive and aggressive adolescents with authoritative and indulgent parents. The outcomes in indulgent families were even better than those in authoritative families, which contradicts the need for strictness and stresses the role of parental warmth in raising aggressive children. A general level of parental control also influences the dynamics of siblings’ relationships, in line with the study of Gozu and Newman [47], whose participants evaluated parental treatment as less fair if they received slightly more control than their siblings.

Generally, our research proved only a certain level of correlation between observed variables, not clear causality. Therefore, there may also be a supporting impact of positively perceived sibling relationships on the raising style of both parents. The interconnectedness of these processes points to the systemic nature of family relationships [2, 51]. Our research does not directly involve the quality of marital relationships, although it generally influences the quality of siblings’ bonds [52]. In correspondence with the systemic approach towards family relations, we presume some symptoms of marital relationship quality in overall parenting styles (as well as the feedback impact of siblings’ relationship characteristics on the marital relationship).

Considering the gender differences between parents, we can observe more statistically significant correlations with siblings’ scales in all dimensions of paternal style. In the dimension of the positive emotional relationship, fathers share with mothers correlations with Admiration by sibling, Affection, Companionship and Prosocial behavior; moreover, their positive style correlates with the Admiration of sibling and Nurturance by sibling. These scales are more typical in younger children. This evokes the idea of fathers’ importance for these children because their involvement in childcare is more crucial in families with more than one child. Generally, we noticed higher level of involvement as well as control of mothers which may reflect a general tendency of mothers to spend more time with children in comparison with fathers. This conclusion was also reached in the study of Axpe [29].

For negative emotional relationships, the correlation with Paternal partiality was the only significant one for mothers. This fact is interesting by itself and may indicate the existence of ‘unhealthy’ family coalitions of one parent with one sibling against the other one with the next sibling/s [51]. Regarding the importance of parental partiality, our findings indicate that siblings in early adulthood appear to be sensitive to differential parental treatment [8]. Parental differentiation may deepen during adulthood [17]. Remarkably, this variable is significant only in the case of fathers. Except for parental partiality, the negative emotional relationship with a father was positively connected with the Quarreling and Antagonism and negatively connected with the Admiration by sibling.

The fact of more correlations in fathers is plausible also for parental control. Whereas the requirement component is connected to Competition and Quarreling by sibling in both parents, fathers ‘succeeded’ in the Paternal partiality, Antagonism, and Domination by sibling. Similarly, the freedom component correlates with Admiration and Nurturance by sibling in the case of both parents. Moreover, the freedom provided by fathers is associated with the Affection and Admiration of sibling. Apparently, paternal emotional relationships, as well as behavioral control, are important for the Admiration of sibling. Thus, the positive perception of a father may coincide with an atmosphere of respect and admiration toward others in a family. Analogically, Antagonism appears to coexist with negative emotional relationships and requirements. Apparently, a negative perception of the father’s role elicits more symptoms of antagonism in siblings’ relationships.

Our observation of the father’s role in the quality of siblings’ relationships contradicts the findings of De Bel, Kalmijn and van Duijn [14], who focused on siblings’ relationships with adults. The authors conclude that regarding support exchange and contact in the family, the mother-child relationship has a bigger impact on siblings than the father-child relationship. This essential difference may be explained by the different age variances in the cited study (18 to 79 years) as well as with different methodologies, following sibling—parent—sibling triads in the form of interviews directed at the present state of mutual relationships. In contrast, our study deals with a retrospective view of parenting styles and the current perception of sibling relationships. Moreover, our respondents often live in the same household as their families, which may substantially modify their perception of relationships.

Generally, our findings on the importance of the parental role in the quality of siblings’ relationships may arise from the developmental specifics of our respondents. Regarding the problem of child anxiety, Verhoeven, Bogels and van der Bruggen [53] determined that especially in older adolescents, paternal overcontrol behavior was more influential than maternal overcontrol for the level of anxiety. Nevertheless, this relationship was weaker among younger adolescents. In agreement with this study, our findings imply the importance of fathers’ role in older adolescents.

Furthermore, we should consider the cultural relativity of our findings and in/congruency in different cultural contexts. The original version of the conception of parenting styles was nested in the Euro-American, specifically the Anglo-Saxon, social environment [24], which showed the optimal parental outcomes of the authoritative parenting style in terms of the highest psychosocial competences and lowest psychological and behavioral dysfunctions [54]. Other studies from different European and Latin American contexts revealed the optimal impact of indulgent style on adjustment even in long-term observation [30–32, 48–50, 55]. Our results are in agreement with these observations, demonstrating positive impact of low strictness on the quality of sibling relationships. On the other hand, research from Arabian countries supports the authoritarian style with high strictness, which seems beneficial in given cultural contexts [56]. These findings highlight the importance of general consistency between culture and optimal parenting style [48]. Nevertheless, studies on parenting, emotional intelligence and mental health among Taiwanese children refer to the negative impact of traditional authoritarian styles on mental health [57]. The relevance of parenting styles should be assessed from the viewpoint of cultural and historical contexts. Shifts in the optimal parenting style paradigm have been observed in various countries [30, 39, 57].

### Limitations and implications for research and practice

The authors of the paper are aware that despite the benefits and potential implications of the findings, this research is not without certain limitations. First, the character of the relationship was reported only subjectively and thus judged from one side of the triad: child-sibling-parents. However, even though objectivity is limited, this perspective plays a prominent role in the development of children and adolescents. Moreover, this has the highest importance and practical implications for our findings. Relying only on respondents’ self-reports of perceived family relations may lead to incomplete information about the family system [58]. However, our intention is not to dwell on the whole family system, but to explore the students’ subjective perception of this system, which is crucial for their personal identity, academic achievement, and well-being.

The second limitation could be seen in the slight imbalance in the gender distribution of the research sample (in accordance with the gender balance among economics students where women prevail). Moreover, the researchers who study parenting styles should be aware of the possible cultural bias because parenting styles are largely influenced by the socio-cultural context [59, 60].

### Future directions for research

The present research provides a unique viewpoint to assess the connections between the characteristics of siblings’ relationships and parental styles. As the starting point for future research in this field, a closer focus on gender differences and age spacing between siblings should be considered. These characteristics were not deliberately incorporated into the data analysis. A further longitudinal study aimed at the development and transformation of the siblings’ relationships during adolescence could be designed, while researchers may build on the current findings and model of interactions proposed higher. The specificity of the father’s role and the impact of his parenting style in families with more than one child has not been closely examined in previous studies. Thus, this issue could also be examined in future research to set the question of parental partiality on siblings’ relationships.

### Implications for practice

This study adds to the body of research that highlights the importance of the parental role to the quality of siblings’ relationships. In terms of practical implications, this suggests emphasizing the whole triad of the family system when dealing with a relationship problem in psychological counseling.

For a specific group of students, we suggest incorporating focused programs into the curriculum, raising awareness of the impact of the functioning of the whole family system on the current behavior of students in their newly built relationship with peers. Understanding the background of their own actions may set a breakpoint in building self-concept and strengthen control over socially positive behaviors.

## Supporting information

S1 Data The original data file for the data analysis. (XLSX)
